# 2,3,5-Triphenyl-2*H*-tetra­zol-3-ium iodide

**DOI:** 10.1107/S1600536812033661

**Published:** 2012-08-01

**Authors:** Hoong-Kun Fun, Tze Shyang Chia, Gamal A. E. Mostafa, Mohamed M. Abunassif, Hatem A. Abdel-Aziz

**Affiliations:** a X-ray Crystallography Unit, School of Physics, Universiti Sains Malaysia, 11800 USM, Penang, Malaysia; b Department of Pharmaceutical Chemistry, College of Pharmacy, King Saud University, PO Box 2457, Riyadh 11451, Saudi Arabia

## Abstract

The asymmetric unit of the title mol­ecular salt, C_19_H_15_N_4_^+^·I^−^, contains four 2,3,5-triphenyl-2*H*-tetra­zol-3-ium cations and five iodide anions, with two of the latter lying on crystallographic inversion centres. In each cation, the tetra­zole ring is essentially planar (r.m.s. deviations = 0.004–0.007 Å). The dihedral angles between the tetra­zole ring and its three attached benzene rings in the four independent cations are: 12.9 (4), 67.0 (4), 48.1 (4); 20.8 (4), 51.1 (4), 62.3 (4); 11.4 (4), 52.3 (4), 47.3 (4) and 6.0 (4), 85.7 (4), 43.5 (4)°. A C—H⋯I hydrogen bond and C—H⋯π inter­actions are observed in the crystal.

## Related literature

For the biological activity of the triphenyl­tetra­zolium ion, see: Mostafa (2007 ▶); Hassanien *et al.* (2003 ▶); Abbas *et al.* (2001 ▶). For the stability of the temperature controller used in the data collection, see: Cosier & Glazer (1986 ▶).
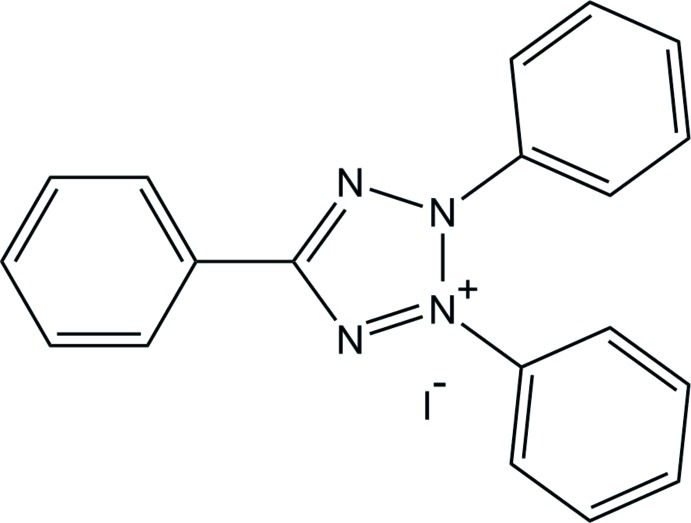


## Experimental

### 

#### Crystal data

C_19_H_15_N_4_^+^·I^−^
*M* *_r_* = 426.25
Monoclinic, 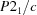

*a* = 9.6541 (4) Å
*b* = 30.9983 (14) Å
*c* = 24.3469 (10) Å
β = 97.930 (1)°
*V* = 7216.4 (5) Å^3^
*Z* = 16
Mo *K*α radiation
μ = 1.78 mm^−1^
*T* = 100 K
0.37 × 0.21 × 0.06 mm

#### Data collection

Bruker APEX DUO CCD diffractometer
Absorption correction: multi-scan (*SADABS*; Bruker, 2009) ▶ *T*_min_ = 0.556, *T*_max_ = 0.907
71406 measured reflections
16492 independent reflections
13800 reflections with *I* > 2σ(*I*)
*R*_int_ = 0.048

#### Refinement

*R*[*F* ^2^ > 2σ(*F* ^2^)] = 0.075
*wR*(*F* ^2^) = 0.172
*S* = 1.26
16492 reflections
868 parameters
H-atom parameters constrained
Δρ_max_ = 5.04 e Å^−3^
Δρ_min_ = −2.28 e Å^−3^

### 

Data collection: *APEX2* (Bruker, 2009) ▶; cell refinement: *SAINT* (Bruker, 2009) ▶; data reduction: *SAINT*; program(s) used to solve structure: *SHELXTL* (Sheldrick, 2008 ▶); program(s) used to refine structure: *SHELXTL*; molecular graphics: *SHELXTL*; software used to prepare material for publication: *SHELXTL* and *PLATON* (Spek, 2009 ▶).

## Supplementary Material

Crystal structure: contains datablock(s) global, I. DOI: 10.1107/S1600536812033661/hb6907sup1.cif

Structure factors: contains datablock(s) I. DOI: 10.1107/S1600536812033661/hb6907Isup2.hkl

Supplementary material file. DOI: 10.1107/S1600536812033661/hb6907Isup3.cml

Additional supplementary materials:  crystallographic information; 3D view; checkCIF report

## Figures and Tables

**Table 1:** Hydrogen-bond geometry (Å, °) *Cg*1, *Cg*2, *Cg*3 and *Cg*4 are the centroids of the C1*C*–C6*C*, C8*C*–C13*C*, C1*A*–C6*A* and C1*D*–C6*D* rings, respectively.

*D*—H⋯*A*	*D*—H	H⋯*A*	*D*⋯*A*	*D*—H⋯*A*
C19*B*—H19*B*⋯I3	0.95	3.03	3.844 (8)	145
C3*A*—H3*AA*⋯*Cg*1^i^	0.95	2.87	3.636 (8)	138
C5*A*—H5*AA*⋯*Cg*2	0.95	2.89	3.547 (9)	127
C16*A*—H16*A*⋯*Cg*3^ii^	0.95	2.85	3.657 (9)	144
C16*D*—H16*D*⋯*Cg*4^iii^	0.95	2.96	3.769 (10)	144

Symmetry codes: (i) 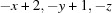; (ii) 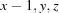; (iii) 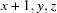.

**Fig. 1.**
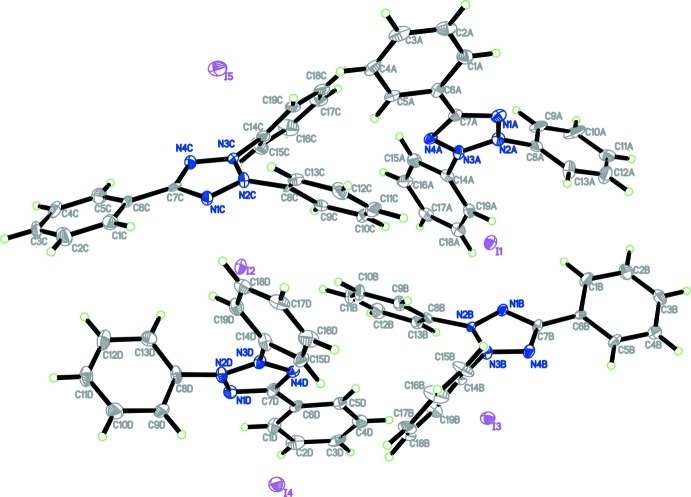
The molecular structure of the title compound with 50% probability displacement ellipsoids.

**Fig. 2.**
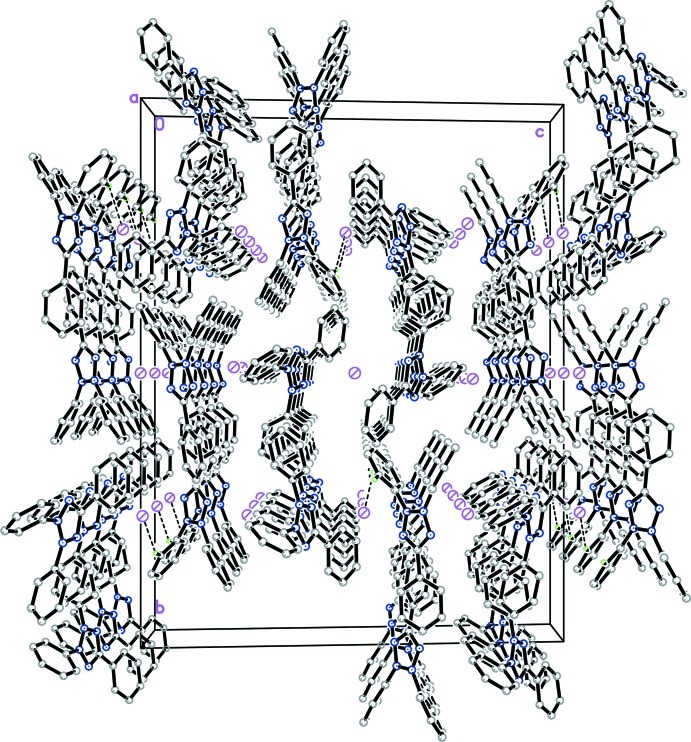
The crystal packing of the title compound. The dashed lines represent the hydrogen bonds. For clarity sake, hydrogen atoms not involved in hydrogen bonding have been omitted.

## supplementary crystallographic information

### Comment

2,3,5-Triphenyltetrazolium ion is used as indicator of bacterial dehydrogenase activity and as a reagent in colorimetric determination method for glucose dehydrogenase. It is also used as ion-pair reagent for determination of antimony in waste water (Mostafa, 2007; Hassanien *et al.*, 2003; Abbas *et al.*, 2001).

The molecular structure of the title compound is shown in Fig. 1. The asymmetric unit of the title compound, C_19_H_15_N_4_^+^, I^-^, contains four crystallographically independent 2,3,5-triphenyl-2*H*-tetrazol-3-ium cations, three fully occupied iodine anions (I1, I2 & I3) and two half-occupied iodine anions (I4 & I5). In the cation *A*, *B*, *C* and *D*, the tetrazole ring [N1–N4/C7] is essentially planar with *r.m.s.* deviations of 0.007, 0.007, 0.006 and 0.004 Å, respectively. The dihedral angles between the tetrazole ring and benzene rings [C1–C6, C8–C13 & C14–C19] are 12.9 (4), 67.0 (4) and 48.1 (4)° in cation *A*, 20.8 (4), 51.1 (4) and 62.3 (4)° in cation *B*, 11.4 (4), 52.3 (4) and 47.3 (4)° in cation *C* and 6.0 (4), 85.7 (4) and 43.5 (4)° in cation *D*.

In the crystal (Fig. 2), intermolecular C19B—H19B···I3 hydrogen bond and C—H···π interactions are observed, involving *Cg*1, *Cg*2, *Cg*3 and *Cg*4 which are the centroids of C1C–C6C, C8C–C13C, C1A–C6A and C1D–C6D rings, respectively.

### Experimental

Upon the addition of triphenyltetrazolium chloride solution (50 ml, 1X10^-2^*M*) to a solution of potassium iodide (50 ml), a yellowish precipitate was formed. The precipitate was filtered off, washed with cold deionized water until no chloride ions were detected in the washing solution. The precipitate was dried under vacuum to give the title ion-pair complex. Orange plates were obtained by slow evaporation of an ethanol solution.

### Refinement

All H atoms were positioned geometrically [C—H = 0.95 Å] and refined using a riding model with *U*_iso_(H) = 1.2*U*_eq_(C). Ten outliers, (1 11 8), (1 16 3), (2 18 5), (0 1 22), (0 12 0), (0 5 22), (1 1 24), (2 7 7), (0 9 18) and (1 1 24) were omitted in the final refinement. The largest difference peak is 1.52 Å from atom I1.

### Crystal data

**Table:**

C_19_H_15_N_4_^+^·I^−^	*F*(000) = 3360
*M**_r_* = 426.25	*D*_x_ = 1.569 Mg m^−^^3^
Monoclinic, *P*2_1_/*c*	Mo *K*α radiation, λ = 0.71073 Å
Hall symbol: -P 2ybc	Cell parameters from 9972 reflections
*a* = 9.6541 (4) Å	θ = 2.9–32.1°
*b* = 30.9983 (14) Å	µ = 1.78 mm^−^^1^
*c* = 24.3469 (10) Å	*T* = 100 K
β = 97.930 (1)°	Plate, orange
*V* = 7216.4 (5) Å^3^	0.37 × 0.21 × 0.06 mm
*Z* = 16	

### Data collection

**Table:**

Bruker APEX DUO CCD diffractometer	16492 independent reflections
Radiation source: fine-focus sealed tube	13800 reflections with *I* > 2σ(*I*)
Graphite monochromator	*R*_int_ = 0.048
φ and ω scans	θ_max_ = 27.5°, θ_min_ = 1.7°
Absorption correction: multi-scan (*SADABS*; Bruker, 2009)	*h* = −12→12
*T*_min_ = 0.556, *T*_max_ = 0.907	*k* = −40→40
71406 measured reflections	*l* = −31→31

### Refinement

**Table:**

Refinement on *F*^2^	Primary atom site location: structure-invariant direct methods
Least-squares matrix: full	Secondary atom site location: difference Fourier map
*R*[*F*^2^ > 2σ(*F*^2^)] = 0.075	Hydrogen site location: inferred from neighbouring sites
*wR*(*F*^2^) = 0.172	H-atom parameters constrained
*S* = 1.26	*w* = 1/[σ^2^(*F*_o_^2^) + (0.0155*P*)^2^ + 127.6*P*] where *P* = (*F*_o_^2^ + 2*F*_c_^2^)/3
16492 reflections	(Δ/σ)_max_ < 0.001
868 parameters	Δρ_max_ = 5.04 e Å^−^^3^
0 restraints	Δρ_min_ = −2.28 e Å^−^^3^

### Special details

**Table:**

Experimental. The crystal was placed in the cold stream of an Oxford Cryosystems Cobra open-flow nitrogen cryostat (Cosier & Glazer, 1986) operating at 100.0 (1) K.
Geometry. All e.s.d.'s (except the e.s.d. in the dihedral angle between two l.s. planes) are estimated using the full covariance matrix. The cell e.s.d.'s are taken into account individually in the estimation of e.s.d.'s in distances, angles and torsion angles; correlations between e.s.d.'s in cell parameters are only used when they are defined by crystal symmetry. An approximate (isotropic) treatment of cell e.s.d.'s is used for estimating e.s.d.'s involving l.s. planes.
Refinement. Refinement of *F*^2^ against ALL reflections. The weighted *R*-factor *wR* and goodness of fit *S* are based on *F*^2^, conventional *R*-factors *R* are based on *F*, with *F* set to zero for negative *F*^2^. The threshold expression of *F*^2^ > σ(*F*^2^) is used only for calculating *R*-factors(gt) *etc*. and is not relevant to the choice of reflections for refinement. *R*-factors based on *F*^2^ are statistically about twice as large as those based on *F*, and *R*- factors based on ALL data will be even larger.

### Fractional atomic coordinates and isotropic or equivalent isotropic displacement parameters (Å^2^)

**Table:**

	*x*	*y*	*z*	*U*_iso_*/*U*_eq_	
I1	0.93818 (5)	0.258521 (17)	0.25097 (2)	0.01657 (12)	
I2	0.57535 (5)	0.489539 (17)	0.22730 (2)	0.01779 (12)	
I3	0.52640 (5)	0.251333 (18)	0.47905 (2)	0.02025 (13)	
I4	1.0000	0.5000	0.5000	0.02196 (17)	
I5	1.0000	0.5000	0.0000	0.0287 (2)	
N1A	0.8507 (6)	0.2267 (2)	0.0944 (3)	0.0170 (14)	
N2A	0.7386 (6)	0.2201 (2)	0.1187 (3)	0.0153 (13)	
N3A	0.6758 (6)	0.2575 (2)	0.1261 (3)	0.0154 (13)	
N4A	0.7434 (6)	0.2896 (2)	0.1059 (3)	0.0147 (13)	
C1A	1.0411 (8)	0.2688 (3)	0.0272 (3)	0.0207 (17)	
H1AA	1.0390	0.2381	0.0275	0.025*	
C2A	1.1314 (8)	0.2904 (3)	−0.0032 (3)	0.0236 (19)	
H2AA	1.1922	0.2747	−0.0233	0.028*	
C3A	1.1321 (8)	0.3348 (3)	−0.0039 (3)	0.0233 (19)	
H3AA	1.1925	0.3495	−0.0253	0.028*	
C4A	1.0467 (9)	0.3586 (3)	0.0260 (3)	0.0216 (18)	
H4AA	1.0494	0.3892	0.0253	0.026*	
C5A	0.9562 (8)	0.3372 (3)	0.0572 (3)	0.0165 (16)	
H5AA	0.8977	0.3533	0.0780	0.020*	
C6A	0.9524 (8)	0.2926 (3)	0.0577 (3)	0.0148 (15)	
C7A	0.8506 (7)	0.2698 (3)	0.0858 (3)	0.0148 (15)	
C8A	0.6944 (8)	0.1775 (3)	0.1328 (3)	0.0172 (16)	
C9A	0.5735 (10)	0.1609 (3)	0.1037 (3)	0.026 (2)	
H9AA	0.5180	0.1773	0.0759	0.032*	
C10A	0.5358 (12)	0.1190 (4)	0.1166 (4)	0.037 (3)	
H10A	0.4523	0.1066	0.0978	0.044*	
C11A	0.6195 (10)	0.0951 (3)	0.1565 (4)	0.027 (2)	
H11A	0.5936	0.0665	0.1649	0.032*	
C12A	0.7401 (10)	0.1130 (3)	0.1841 (4)	0.029 (2)	
H12A	0.7968	0.0966	0.2115	0.034*	
C13A	0.7799 (9)	0.1545 (3)	0.1723 (4)	0.0241 (18)	
H13A	0.8637	0.1667	0.1909	0.029*	
C14A	0.5483 (7)	0.2627 (3)	0.1496 (3)	0.0147 (15)	
C15A	0.4442 (8)	0.2876 (3)	0.1199 (4)	0.0219 (18)	
H15A	0.4576	0.3011	0.0860	0.026*	
C16A	0.3204 (9)	0.2921 (3)	0.1418 (3)	0.0231 (18)	
H16A	0.2477	0.3094	0.1229	0.028*	
C17A	0.3004 (8)	0.2720 (3)	0.1905 (3)	0.0203 (17)	
H17A	0.2132	0.2747	0.2042	0.024*	
C18A	0.4077 (8)	0.2476 (3)	0.2201 (3)	0.0195 (17)	
H18A	0.3943	0.2342	0.2541	0.023*	
C19A	0.5340 (8)	0.2429 (3)	0.1994 (3)	0.0173 (16)	
H19A	0.6084	0.2266	0.2189	0.021*	
N1B	0.6359 (6)	0.2296 (2)	0.3374 (3)	0.0138 (13)	
N2B	0.6802 (6)	0.2683 (2)	0.3522 (2)	0.0115 (12)	
N3B	0.8016 (6)	0.2657 (2)	0.3865 (3)	0.0157 (13)	
N4B	0.8386 (7)	0.2251 (2)	0.3944 (3)	0.0151 (13)	
C1B	0.6439 (9)	0.1366 (3)	0.3153 (3)	0.0222 (18)	
H1BA	0.5938	0.1535	0.2867	0.027*	
C2B	0.6349 (10)	0.0917 (3)	0.3136 (4)	0.0254 (19)	
H2BA	0.5765	0.0782	0.2839	0.030*	
C3B	0.7092 (9)	0.0668 (3)	0.3539 (4)	0.0231 (18)	
H3BA	0.7038	0.0363	0.3516	0.028*	
C4B	0.7924 (9)	0.0864 (3)	0.3981 (4)	0.0244 (19)	
H4BA	0.8420	0.0693	0.4267	0.029*	
C5B	0.8022 (9)	0.1307 (3)	0.4001 (3)	0.0209 (17)	
H5BA	0.8614	0.1440	0.4297	0.025*	
C6B	0.7271 (8)	0.1562 (3)	0.3596 (3)	0.0122 (14)	
C7B	0.7345 (7)	0.2032 (3)	0.3648 (3)	0.0131 (14)	
C8B	0.6083 (8)	0.3072 (3)	0.3318 (3)	0.0150 (15)	
C9B	0.6795 (9)	0.3391 (3)	0.3069 (3)	0.0201 (17)	
H9BA	0.7771	0.3369	0.3055	0.024*	
C10B	0.6032 (10)	0.3740 (3)	0.2844 (4)	0.0248 (19)	
H10B	0.6485	0.3964	0.2671	0.030*	
C11B	0.4610 (10)	0.3766 (3)	0.2870 (4)	0.0250 (19)	
H11B	0.4099	0.4009	0.2712	0.030*	
C12B	0.3925 (9)	0.3448 (3)	0.3120 (4)	0.0246 (19)	
H12B	0.2949	0.3471	0.3132	0.030*	
C13B	0.4661 (8)	0.3090 (3)	0.3357 (3)	0.0176 (16)	
H13B	0.4210	0.2870	0.3538	0.021*	
C14B	0.8811 (8)	0.3016 (3)	0.4122 (3)	0.0164 (16)	
C15B	1.0164 (9)	0.3075 (3)	0.3998 (4)	0.027 (2)	
H15B	1.0562	0.2883	0.3760	0.032*	
C16B	1.0896 (9)	0.3421 (4)	0.4234 (4)	0.032 (2)	
H16B	1.1821	0.3472	0.4158	0.039*	
C17B	1.0297 (9)	0.3702 (3)	0.4586 (3)	0.0241 (19)	
H17B	1.0807	0.3946	0.4738	0.029*	
C18B	0.8969 (9)	0.3625 (3)	0.4713 (3)	0.0210 (17)	
H18B	0.8577	0.3812	0.4959	0.025*	
C19B	0.8205 (8)	0.3274 (3)	0.4481 (3)	0.0197 (17)	
H19B	0.7295	0.3215	0.4568	0.024*	
N1C	0.8767 (7)	0.5227 (2)	0.1441 (3)	0.0156 (13)	
N2C	0.8347 (7)	0.4841 (2)	0.1274 (3)	0.0158 (13)	
N3C	0.7115 (7)	0.4867 (2)	0.0944 (3)	0.0140 (13)	
N4C	0.6703 (7)	0.5273 (2)	0.0900 (3)	0.0165 (14)	
C1C	0.8953 (9)	0.6160 (3)	0.1564 (3)	0.0211 (17)	
H1CA	0.9776	0.5996	0.1676	0.025*	
C2C	0.8951 (10)	0.6600 (3)	0.1653 (4)	0.028 (2)	
H2CA	0.9771	0.6739	0.1829	0.033*	
C3C	0.7753 (10)	0.6838 (3)	0.1485 (4)	0.0243 (19)	
H3CA	0.7757	0.7142	0.1540	0.029*	
C4C	0.6547 (10)	0.6635 (3)	0.1236 (4)	0.0247 (19)	
H4CA	0.5726	0.6800	0.1126	0.030*	
C5C	0.6532 (9)	0.6196 (3)	0.1146 (4)	0.0224 (18)	
H5CA	0.5704	0.6058	0.0975	0.027*	
C6C	0.7737 (8)	0.5955 (3)	0.1308 (3)	0.0157 (15)	
C7C	0.7730 (8)	0.5489 (3)	0.1213 (3)	0.0146 (15)	
C8C	0.9108 (9)	0.4455 (3)	0.1465 (3)	0.0155 (15)	
C9C	0.8409 (10)	0.4135 (3)	0.1721 (3)	0.0215 (18)	
H9CA	0.7437	0.4156	0.1744	0.026*	
C10C	0.9187 (11)	0.3784 (3)	0.1941 (4)	0.027 (2)	
H10C	0.8738	0.3554	0.2105	0.033*	
C11C	1.0619 (10)	0.3766 (3)	0.1923 (4)	0.029 (2)	
H11C	1.1147	0.3529	0.2086	0.035*	
C12C	1.1282 (10)	0.4091 (3)	0.1668 (4)	0.028 (2)	
H12C	1.2258	0.4073	0.1651	0.034*	
C13C	1.0522 (9)	0.4445 (3)	0.1437 (3)	0.0201 (17)	
H13C	1.0966	0.4673	0.1266	0.024*	
C14C	0.6317 (8)	0.4508 (3)	0.0688 (3)	0.0161 (16)	
C15C	0.4901 (9)	0.4498 (3)	0.0739 (4)	0.027 (2)	
H15C	0.4465	0.4725	0.0914	0.033*	
C16C	0.4160 (10)	0.4139 (4)	0.0520 (4)	0.032 (2)	
H16C	0.3195	0.4114	0.0555	0.038*	
C17C	0.4797 (9)	0.3819 (3)	0.0254 (3)	0.0245 (19)	
H17C	0.4272	0.3576	0.0108	0.029*	
C18C	0.6188 (9)	0.3850 (3)	0.0198 (3)	0.0227 (18)	
H18C	0.6620	0.3627	0.0016	0.027*	
C19C	0.6970 (9)	0.4203 (3)	0.0404 (3)	0.0193 (17)	
H19C	0.7921	0.4234	0.0351	0.023*	
N1D	0.6524 (6)	0.5202 (2)	0.3844 (3)	0.0157 (13)	
N2D	0.7624 (6)	0.5296 (2)	0.3600 (3)	0.0161 (14)	
N3D	0.8316 (6)	0.4933 (2)	0.3514 (3)	0.0142 (13)	
N4D	0.7692 (6)	0.4598 (2)	0.3707 (3)	0.0141 (13)	
C1D	0.4534 (8)	0.4737 (3)	0.4414 (3)	0.0205 (17)	
H1DA	0.4416	0.5040	0.4367	0.025*	
C2D	0.3652 (8)	0.4506 (3)	0.4708 (4)	0.0238 (19)	
H2DA	0.2926	0.4649	0.4862	0.029*	
C3D	0.3834 (8)	0.4068 (3)	0.4776 (3)	0.0222 (19)	
H3DA	0.3241	0.3912	0.4986	0.027*	
C4D	0.4863 (9)	0.3849 (3)	0.4545 (3)	0.0222 (18)	
H4DA	0.4972	0.3546	0.4592	0.027*	
C5D	0.5737 (8)	0.4080 (3)	0.4242 (3)	0.0186 (16)	
H5DA	0.6433	0.3934	0.4072	0.022*	
C6D	0.5585 (8)	0.4527 (3)	0.4188 (3)	0.0160 (16)	
C7D	0.6582 (8)	0.4777 (3)	0.3911 (3)	0.0164 (16)	
C8D	0.8017 (8)	0.5739 (3)	0.3502 (4)	0.0186 (17)	
C9D	0.8910 (9)	0.5946 (3)	0.3897 (3)	0.0225 (18)	
H9DA	0.9335	0.5801	0.4221	0.027*	
C10D	0.9173 (11)	0.6380 (3)	0.3806 (4)	0.030 (2)	
H10D	0.9802	0.6534	0.4071	0.036*	
C11D	0.8547 (9)	0.6591 (3)	0.3342 (4)	0.0262 (19)	
H11D	0.8722	0.6889	0.3295	0.031*	
C12D	0.7651 (9)	0.6369 (3)	0.2938 (4)	0.027 (2)	
H12D	0.7231	0.6516	0.2614	0.033*	
C13D	0.7376 (9)	0.5937 (3)	0.3007 (4)	0.0234 (18)	
H13D	0.6782	0.5780	0.2734	0.028*	
C14D	0.9600 (8)	0.4902 (3)	0.3280 (4)	0.0182 (16)	
C15D	1.0620 (9)	0.4632 (3)	0.3549 (3)	0.0213 (18)	
H15D	1.0481	0.4479	0.3876	0.026*	
C16D	1.1867 (9)	0.4595 (3)	0.3318 (4)	0.027 (2)	
H16D	1.2583	0.4410	0.3488	0.032*	
C17D	1.2077 (9)	0.4822 (3)	0.2849 (4)	0.0240 (19)	
H17D	1.2935	0.4796	0.2702	0.029*	
C18D	1.1012 (8)	0.5089 (3)	0.2594 (3)	0.0211 (17)	
H18D	1.1152	0.5245	0.2270	0.025*	
C19D	0.9749 (8)	0.5131 (3)	0.2805 (4)	0.0206 (17)	
H19D	0.9021	0.5310	0.2629	0.025*	

### Atomic displacement parameters (Å^2^)

**Table:**

	*U*^11^	*U*^22^	*U*^33^	*U*^12^	*U*^13^	*U*^23^
I1	0.0169 (2)	0.0156 (3)	0.0177 (2)	0.00108 (19)	0.00432 (18)	0.0040 (2)
I2	0.0174 (2)	0.0146 (3)	0.0220 (3)	0.0025 (2)	0.00503 (19)	0.0049 (2)
I3	0.0233 (3)	0.0208 (3)	0.0167 (2)	−0.0055 (2)	0.00297 (19)	0.0000 (2)
I4	0.0230 (4)	0.0236 (4)	0.0189 (4)	0.0050 (3)	0.0015 (3)	−0.0013 (3)
I5	0.0431 (5)	0.0245 (4)	0.0181 (4)	−0.0207 (4)	0.0031 (3)	−0.0041 (3)
N1A	0.012 (3)	0.023 (4)	0.016 (3)	0.000 (3)	0.005 (2)	0.004 (3)
N2A	0.012 (3)	0.017 (4)	0.018 (3)	0.000 (3)	0.006 (2)	0.002 (3)
N3A	0.015 (3)	0.012 (3)	0.019 (3)	−0.001 (3)	0.001 (2)	0.002 (3)
N4A	0.015 (3)	0.018 (4)	0.012 (3)	−0.001 (3)	0.006 (2)	0.004 (3)
C1A	0.019 (4)	0.025 (5)	0.018 (4)	0.001 (3)	0.004 (3)	0.000 (3)
C2A	0.017 (4)	0.038 (6)	0.019 (4)	−0.001 (4)	0.010 (3)	0.000 (4)
C3A	0.012 (3)	0.040 (6)	0.017 (4)	−0.005 (4)	0.002 (3)	0.006 (4)
C4A	0.024 (4)	0.023 (5)	0.016 (4)	−0.007 (3)	−0.004 (3)	0.007 (3)
C5A	0.016 (3)	0.023 (4)	0.010 (3)	−0.001 (3)	0.002 (3)	0.003 (3)
C6A	0.012 (3)	0.015 (4)	0.017 (4)	−0.002 (3)	0.001 (3)	0.003 (3)
C7A	0.012 (3)	0.019 (4)	0.014 (4)	0.000 (3)	0.005 (3)	0.003 (3)
C8A	0.024 (4)	0.016 (4)	0.014 (4)	0.000 (3)	0.010 (3)	0.000 (3)
C9A	0.041 (5)	0.027 (5)	0.011 (4)	−0.015 (4)	0.002 (3)	0.003 (3)
C10A	0.053 (6)	0.041 (6)	0.014 (4)	−0.024 (5)	−0.001 (4)	0.008 (4)
C11A	0.047 (5)	0.019 (4)	0.018 (4)	−0.011 (4)	0.015 (4)	−0.001 (3)
C12A	0.034 (5)	0.020 (5)	0.031 (5)	0.002 (4)	0.000 (4)	0.008 (4)
C13A	0.020 (4)	0.017 (4)	0.034 (5)	0.001 (3)	0.001 (3)	0.004 (4)
C14A	0.009 (3)	0.018 (4)	0.017 (4)	−0.002 (3)	0.004 (3)	−0.005 (3)
C15A	0.019 (4)	0.024 (5)	0.022 (4)	0.005 (3)	0.004 (3)	−0.001 (4)
C16A	0.021 (4)	0.031 (5)	0.017 (4)	0.004 (4)	0.003 (3)	−0.001 (4)
C17A	0.015 (4)	0.023 (5)	0.023 (4)	−0.003 (3)	0.006 (3)	−0.007 (4)
C18A	0.019 (4)	0.017 (4)	0.024 (4)	−0.009 (3)	0.010 (3)	−0.002 (3)
C19A	0.016 (3)	0.015 (4)	0.022 (4)	0.001 (3)	0.005 (3)	0.000 (3)
N1B	0.016 (3)	0.011 (3)	0.014 (3)	−0.001 (3)	0.001 (2)	0.003 (3)
N2B	0.017 (3)	0.009 (3)	0.009 (3)	0.000 (2)	0.002 (2)	0.003 (2)
N3B	0.012 (3)	0.014 (3)	0.021 (3)	−0.001 (3)	0.000 (2)	−0.001 (3)
N4B	0.016 (3)	0.013 (3)	0.017 (3)	0.000 (3)	0.005 (2)	0.004 (3)
C1B	0.026 (4)	0.020 (4)	0.019 (4)	−0.001 (4)	−0.003 (3)	0.006 (3)
C2B	0.034 (5)	0.015 (4)	0.027 (5)	−0.006 (4)	0.002 (4)	−0.003 (4)
C3B	0.030 (4)	0.016 (4)	0.026 (4)	0.000 (4)	0.014 (4)	−0.003 (4)
C4B	0.032 (5)	0.024 (5)	0.018 (4)	0.008 (4)	0.005 (3)	0.003 (4)
C5B	0.026 (4)	0.021 (4)	0.014 (4)	0.003 (3)	−0.003 (3)	0.006 (3)
C6B	0.015 (3)	0.015 (4)	0.008 (3)	0.000 (3)	0.006 (3)	0.002 (3)
C7B	0.012 (3)	0.013 (4)	0.015 (3)	0.000 (3)	0.002 (3)	0.004 (3)
C8B	0.020 (4)	0.010 (4)	0.013 (4)	0.004 (3)	−0.001 (3)	−0.004 (3)
C9B	0.023 (4)	0.016 (4)	0.022 (4)	0.002 (3)	0.005 (3)	0.001 (3)
C10B	0.038 (5)	0.012 (4)	0.025 (4)	0.004 (4)	0.009 (4)	0.005 (3)
C11B	0.039 (5)	0.013 (4)	0.023 (4)	0.010 (4)	0.002 (4)	0.002 (3)
C12B	0.019 (4)	0.026 (5)	0.028 (5)	0.007 (4)	−0.002 (3)	−0.003 (4)
C13B	0.017 (4)	0.017 (4)	0.018 (4)	0.001 (3)	0.001 (3)	−0.001 (3)
C14B	0.013 (3)	0.016 (4)	0.019 (4)	−0.004 (3)	0.001 (3)	−0.001 (3)
C15B	0.024 (4)	0.033 (5)	0.027 (5)	−0.008 (4)	0.011 (4)	−0.018 (4)
C16B	0.021 (4)	0.046 (6)	0.031 (5)	−0.020 (4)	0.011 (4)	−0.015 (5)
C17B	0.027 (4)	0.026 (5)	0.018 (4)	−0.008 (4)	−0.005 (3)	−0.008 (4)
C18B	0.024 (4)	0.025 (5)	0.014 (4)	−0.002 (4)	0.004 (3)	−0.006 (3)
C19B	0.015 (4)	0.022 (4)	0.024 (4)	−0.003 (3)	0.006 (3)	−0.006 (3)
N1C	0.018 (3)	0.012 (3)	0.017 (3)	0.000 (3)	0.003 (2)	0.001 (3)
N2C	0.016 (3)	0.011 (3)	0.021 (3)	0.000 (3)	0.001 (3)	0.001 (3)
N3C	0.020 (3)	0.012 (3)	0.010 (3)	0.000 (3)	0.001 (2)	0.001 (3)
N4C	0.019 (3)	0.013 (3)	0.017 (3)	0.003 (3)	0.000 (3)	0.001 (3)
C1C	0.022 (4)	0.021 (4)	0.020 (4)	0.003 (3)	0.007 (3)	0.001 (3)
C2C	0.032 (5)	0.022 (5)	0.031 (5)	−0.005 (4)	0.008 (4)	−0.004 (4)
C3C	0.041 (5)	0.009 (4)	0.027 (5)	0.000 (4)	0.022 (4)	0.000 (3)
C4C	0.032 (5)	0.020 (5)	0.025 (4)	0.009 (4)	0.015 (4)	0.011 (4)
C5C	0.022 (4)	0.022 (5)	0.022 (4)	0.002 (3)	0.002 (3)	0.001 (4)
C6C	0.024 (4)	0.013 (4)	0.012 (3)	−0.001 (3)	0.008 (3)	0.003 (3)
C7C	0.020 (4)	0.018 (4)	0.007 (3)	−0.002 (3)	0.006 (3)	0.004 (3)
C8C	0.029 (4)	0.011 (4)	0.007 (3)	0.003 (3)	0.002 (3)	0.005 (3)
C9C	0.035 (5)	0.018 (4)	0.013 (4)	0.002 (4)	0.007 (3)	−0.001 (3)
C10C	0.050 (6)	0.018 (5)	0.017 (4)	0.010 (4)	0.012 (4)	0.006 (3)
C11C	0.040 (5)	0.029 (5)	0.017 (4)	0.017 (4)	0.001 (4)	0.004 (4)
C12C	0.032 (5)	0.026 (5)	0.026 (5)	0.010 (4)	0.001 (4)	−0.006 (4)
C13C	0.027 (4)	0.021 (4)	0.011 (4)	0.001 (3)	−0.001 (3)	0.000 (3)
C14C	0.020 (4)	0.017 (4)	0.010 (3)	−0.003 (3)	−0.001 (3)	0.000 (3)
C15C	0.022 (4)	0.028 (5)	0.032 (5)	−0.004 (4)	0.004 (4)	−0.009 (4)
C16C	0.024 (4)	0.041 (6)	0.030 (5)	−0.013 (4)	0.003 (4)	−0.009 (4)
C17C	0.031 (4)	0.029 (5)	0.012 (4)	−0.013 (4)	−0.003 (3)	−0.004 (3)
C18C	0.033 (5)	0.023 (5)	0.010 (4)	−0.004 (4)	−0.001 (3)	0.000 (3)
C19C	0.025 (4)	0.021 (4)	0.011 (4)	−0.002 (3)	0.002 (3)	−0.002 (3)
N1D	0.015 (3)	0.017 (4)	0.017 (3)	0.000 (3)	0.008 (2)	0.000 (3)
N2D	0.014 (3)	0.012 (3)	0.023 (3)	0.001 (3)	0.005 (3)	0.001 (3)
N3D	0.013 (3)	0.014 (3)	0.016 (3)	−0.001 (3)	0.005 (2)	0.003 (3)
N4D	0.015 (3)	0.016 (3)	0.012 (3)	0.000 (3)	0.003 (2)	0.001 (3)
C1D	0.017 (4)	0.020 (4)	0.025 (4)	0.000 (3)	0.005 (3)	0.004 (3)
C2D	0.011 (3)	0.040 (6)	0.022 (4)	−0.002 (3)	0.008 (3)	0.000 (4)
C3D	0.019 (4)	0.035 (5)	0.013 (4)	−0.012 (4)	0.002 (3)	0.006 (4)
C4D	0.027 (4)	0.023 (5)	0.018 (4)	−0.009 (4)	0.008 (3)	0.000 (3)
C5D	0.023 (4)	0.019 (4)	0.015 (4)	0.001 (3)	0.004 (3)	0.001 (3)
C6D	0.017 (4)	0.019 (4)	0.013 (3)	−0.003 (3)	0.004 (3)	0.006 (3)
C7D	0.014 (3)	0.017 (4)	0.018 (4)	0.001 (3)	0.000 (3)	0.003 (3)
C8D	0.016 (4)	0.011 (4)	0.031 (4)	0.001 (3)	0.010 (3)	0.000 (3)
C9D	0.032 (4)	0.022 (5)	0.015 (4)	−0.005 (4)	0.008 (3)	0.004 (3)
C10D	0.041 (5)	0.026 (5)	0.023 (4)	−0.010 (4)	0.005 (4)	−0.003 (4)
C11D	0.031 (5)	0.016 (4)	0.036 (5)	0.001 (4)	0.020 (4)	0.004 (4)
C12D	0.028 (4)	0.020 (5)	0.036 (5)	0.007 (4)	0.011 (4)	0.008 (4)
C13D	0.026 (4)	0.024 (5)	0.022 (4)	0.002 (4)	0.007 (3)	−0.001 (4)
C14D	0.012 (3)	0.016 (4)	0.028 (4)	0.000 (3)	0.006 (3)	−0.001 (3)
C15D	0.022 (4)	0.023 (5)	0.019 (4)	0.008 (3)	0.006 (3)	0.004 (3)
C16D	0.019 (4)	0.036 (6)	0.025 (4)	0.009 (4)	0.001 (3)	−0.006 (4)
C17D	0.020 (4)	0.031 (5)	0.022 (4)	−0.004 (4)	0.006 (3)	−0.009 (4)
C18D	0.022 (4)	0.022 (5)	0.020 (4)	−0.003 (3)	0.005 (3)	−0.004 (3)
C19D	0.017 (4)	0.017 (4)	0.027 (4)	−0.001 (3)	0.004 (3)	−0.002 (4)

### Geometric parameters (Å, º)

**Table:**

N1A—N2A	1.318 (9)	N1C—N2C	1.310 (9)
N1A—C7A	1.353 (11)	N1C—C7C	1.348 (10)
N2A—N3A	1.333 (9)	N2C—N3C	1.342 (9)
N2A—C8A	1.442 (11)	N2C—C8C	1.448 (10)
N3A—N4A	1.322 (9)	N3C—N4C	1.320 (9)
N3A—C14A	1.437 (9)	N3C—C14C	1.447 (10)
N4A—C7A	1.350 (10)	N4C—C7C	1.343 (10)
C1A—C2A	1.392 (12)	C1C—C2C	1.382 (13)
C1A—C6A	1.416 (11)	C1C—C6C	1.403 (12)
C1A—H1AA	0.9500	C1C—H1CA	0.9500
C2A—C3A	1.375 (14)	C2C—C3C	1.386 (13)
C2A—H2AA	0.9500	C2C—H2CA	0.9500
C3A—C4A	1.384 (13)	C3C—C4C	1.388 (13)
C3A—H3AA	0.9500	C3C—H3CA	0.9500
C4A—C5A	1.402 (11)	C4C—C5C	1.379 (13)
C4A—H4AA	0.9500	C4C—H4CA	0.9500
C5A—C6A	1.382 (12)	C5C—C6C	1.394 (11)
C5A—H5AA	0.9500	C5C—H5CA	0.9500
C6A—C7A	1.457 (10)	C6C—C7C	1.462 (11)
C8A—C13A	1.378 (12)	C8C—C13C	1.376 (11)
C8A—C9A	1.380 (12)	C8C—C9C	1.395 (12)
C9A—C10A	1.397 (13)	C9C—C10C	1.386 (12)
C9A—H9AA	0.9500	C9C—H9CA	0.9500
C10A—C11A	1.389 (14)	C10C—C11C	1.391 (13)
C10A—H10A	0.9500	C10C—H10C	0.9500
C11A—C12A	1.378 (13)	C11C—C12C	1.384 (14)
C11A—H11A	0.9500	C11C—H11C	0.9500
C12A—C13A	1.383 (13)	C12C—C13C	1.396 (12)
C12A—H12A	0.9500	C12C—H12C	0.9500
C13A—H13A	0.9500	C13C—H13C	0.9500
C14A—C19A	1.381 (11)	C14C—C19C	1.373 (12)
C14A—C15A	1.390 (11)	C14C—C15C	1.390 (11)
C15A—C16A	1.381 (11)	C15C—C16C	1.390 (13)
C15A—H15A	0.9500	C15C—H15C	0.9500
C16A—C17A	1.377 (12)	C16C—C17C	1.375 (14)
C16A—H16A	0.9500	C16C—H16C	0.9500
C17A—C18A	1.400 (12)	C17C—C18C	1.371 (12)
C17A—H17A	0.9500	C17C—H17C	0.9500
C18A—C19A	1.389 (11)	C18C—C19C	1.384 (12)
C18A—H18A	0.9500	C18C—H18C	0.9500
C19A—H19A	0.9500	C19C—H19C	0.9500
N1B—N2B	1.307 (9)	N1D—N2D	1.318 (9)
N1B—C7B	1.357 (9)	N1D—C7D	1.328 (11)
N2B—N3B	1.344 (8)	N2D—N3D	1.338 (9)
N2B—C8B	1.446 (10)	N2D—C8D	1.452 (10)
N3B—N4B	1.317 (9)	N3D—N4D	1.319 (9)
N3B—C14B	1.443 (10)	N3D—C14D	1.438 (9)
N4B—C7B	1.337 (10)	N4D—C7D	1.361 (10)
C1B—C6B	1.393 (11)	C1D—C6D	1.383 (11)
C1B—C2B	1.393 (12)	C1D—C2D	1.386 (11)
C1B—H1BA	0.9500	C1D—H1DA	0.9500
C2B—C3B	1.372 (13)	C2D—C3D	1.377 (14)
C2B—H2BA	0.9500	C2D—H2DA	0.9500
C3B—C4B	1.390 (13)	C3D—C4D	1.385 (13)
C3B—H3BA	0.9500	C3D—H3DA	0.9500
C4B—C5B	1.379 (13)	C4D—C5D	1.393 (11)
C4B—H4BA	0.9500	C4D—H4DA	0.9500
C5B—C6B	1.388 (10)	C5D—C6D	1.396 (12)
C5B—H5BA	0.9500	C5D—H5DA	0.9500
C6B—C7B	1.463 (11)	C6D—C7D	1.470 (11)
C8B—C9B	1.389 (12)	C8D—C9D	1.362 (12)
C8B—C13B	1.390 (11)	C8D—C13D	1.417 (12)
C9B—C10B	1.379 (12)	C9D—C10D	1.393 (13)
C9B—H9BA	0.9500	C9D—H9DA	0.9500
C10B—C11B	1.385 (13)	C10D—C11D	1.370 (13)
C10B—H10B	0.9500	C10D—H10D	0.9500
C11B—C12B	1.376 (13)	C11D—C12D	1.399 (14)
C11B—H11B	0.9500	C11D—H11D	0.9500
C12B—C13B	1.398 (12)	C12D—C13D	1.378 (13)
C12B—H12B	0.9500	C12D—H12D	0.9500
C13B—H13B	0.9500	C13D—H13D	0.9500
C14B—C19B	1.374 (11)	C14D—C19D	1.382 (12)
C14B—C15B	1.392 (11)	C14D—C15D	1.387 (11)
C15B—C16B	1.366 (13)	C15D—C16D	1.402 (12)
C15B—H15B	0.9500	C15D—H15D	0.9500
C16B—C17B	1.401 (13)	C16D—C17D	1.378 (13)
C16B—H16B	0.9500	C16D—H16D	0.9500
C17B—C18B	1.379 (12)	C17D—C18D	1.398 (12)
C17B—H17B	0.9500	C17D—H17D	0.9500
C18B—C19B	1.390 (12)	C18D—C19D	1.392 (11)
C18B—H18B	0.9500	C18D—H18D	0.9500
C19B—H19B	0.9500	C19D—H19D	0.9500
			
N2A—N1A—C7A	103.9 (6)	N2C—N1C—C7C	104.0 (6)
N1A—N2A—N3A	110.0 (6)	N1C—N2C—N3C	110.0 (6)
N1A—N2A—C8A	122.5 (7)	N1C—N2C—C8C	122.3 (6)
N3A—N2A—C8A	127.4 (6)	N3C—N2C—C8C	127.6 (7)
N4A—N3A—N2A	110.3 (6)	N4C—N3C—N2C	109.8 (6)
N4A—N3A—C14A	124.0 (7)	N4C—N3C—C14C	124.2 (6)
N2A—N3A—C14A	125.6 (7)	N2C—N3C—C14C	126.0 (7)
N3A—N4A—C7A	103.7 (7)	N3C—N4C—C7C	103.8 (6)
C2A—C1A—C6A	119.6 (8)	C2C—C1C—C6C	119.9 (8)
C2A—C1A—H1AA	120.2	C2C—C1C—H1CA	120.1
C6A—C1A—H1AA	120.2	C6C—C1C—H1CA	120.1
C3A—C2A—C1A	119.6 (8)	C1C—C2C—C3C	119.9 (9)
C3A—C2A—H2AA	120.2	C1C—C2C—H2CA	120.0
C1A—C2A—H2AA	120.2	C3C—C2C—H2CA	120.0
C2A—C3A—C4A	121.5 (8)	C2C—C3C—C4C	120.2 (8)
C2A—C3A—H3AA	119.3	C2C—C3C—H3CA	119.9
C4A—C3A—H3AA	119.3	C4C—C3C—H3CA	119.9
C3A—C4A—C5A	119.6 (9)	C5C—C4C—C3C	120.5 (8)
C3A—C4A—H4AA	120.2	C5C—C4C—H4CA	119.8
C5A—C4A—H4AA	120.2	C3C—C4C—H4CA	119.8
C6A—C5A—C4A	119.7 (8)	C4C—C5C—C6C	119.7 (8)
C6A—C5A—H5AA	120.1	C4C—C5C—H5CA	120.2
C4A—C5A—H5AA	120.1	C6C—C5C—H5CA	120.2
C5A—C6A—C1A	120.0 (7)	C5C—C6C—C1C	119.8 (8)
C5A—C6A—C7A	120.6 (7)	C5C—C6C—C7C	119.9 (7)
C1A—C6A—C7A	119.3 (7)	C1C—C6C—C7C	120.2 (7)
N4A—C7A—N1A	112.1 (7)	N4C—C7C—N1C	112.5 (7)
N4A—C7A—C6A	123.4 (7)	N4C—C7C—C6C	124.7 (7)
N1A—C7A—C6A	124.5 (7)	N1C—C7C—C6C	122.8 (7)
C13A—C8A—C9A	123.2 (8)	C13C—C8C—C9C	123.4 (8)
C13A—C8A—N2A	118.2 (7)	C13C—C8C—N2C	117.7 (7)
C9A—C8A—N2A	118.4 (7)	C9C—C8C—N2C	118.4 (7)
C8A—C9A—C10A	117.4 (9)	C10C—C9C—C8C	117.4 (8)
C8A—C9A—H9AA	121.3	C10C—C9C—H9CA	121.3
C10A—C9A—H9AA	121.3	C8C—C9C—H9CA	121.3
C11A—C10A—C9A	120.5 (9)	C9C—C10C—C11C	120.5 (9)
C11A—C10A—H10A	119.7	C9C—C10C—H10C	119.8
C9A—C10A—H10A	119.7	C11C—C10C—H10C	119.8
C12A—C11A—C10A	119.9 (9)	C12C—C11C—C10C	120.6 (8)
C12A—C11A—H11A	120.1	C12C—C11C—H11C	119.7
C10A—C11A—H11A	120.1	C10C—C11C—H11C	119.7
C11A—C12A—C13A	120.9 (9)	C11C—C12C—C13C	120.2 (9)
C11A—C12A—H12A	119.6	C11C—C12C—H12C	119.9
C13A—C12A—H12A	119.6	C13C—C12C—H12C	119.9
C8A—C13A—C12A	118.1 (8)	C8C—C13C—C12C	117.9 (8)
C8A—C13A—H13A	121.0	C8C—C13C—H13C	121.1
C12A—C13A—H13A	121.0	C12C—C13C—H13C	121.1
C19A—C14A—C15A	123.5 (7)	C19C—C14C—C15C	123.8 (8)
C19A—C14A—N3A	119.7 (7)	C19C—C14C—N3C	119.5 (7)
C15A—C14A—N3A	116.9 (7)	C15C—C14C—N3C	116.7 (7)
C16A—C15A—C14A	117.2 (8)	C14C—C15C—C16C	116.3 (9)
C16A—C15A—H15A	121.4	C14C—C15C—H15C	121.8
C14A—C15A—H15A	121.4	C16C—C15C—H15C	121.8
C17A—C16A—C15A	121.2 (8)	C17C—C16C—C15C	121.2 (9)
C17A—C16A—H16A	119.4	C17C—C16C—H16C	119.4
C15A—C16A—H16A	119.4	C15C—C16C—H16C	119.4
C16A—C17A—C18A	120.4 (8)	C18C—C17C—C16C	120.2 (8)
C16A—C17A—H17A	119.8	C18C—C17C—H17C	119.9
C18A—C17A—H17A	119.8	C16C—C17C—H17C	119.9
C19A—C18A—C17A	119.7 (8)	C17C—C18C—C19C	120.8 (9)
C19A—C18A—H18A	120.2	C17C—C18C—H18C	119.6
C17A—C18A—H18A	120.2	C19C—C18C—H18C	119.6
C14A—C19A—C18A	118.0 (7)	C14C—C19C—C18C	117.4 (8)
C14A—C19A—H19A	121.0	C14C—C19C—H19C	121.3
C18A—C19A—H19A	121.0	C18C—C19C—H19C	121.3
N2B—N1B—C7B	103.7 (6)	N2D—N1D—C7D	104.6 (6)
N1B—N2B—N3B	110.0 (6)	N1D—N2D—N3D	109.6 (6)
N1B—N2B—C8B	123.2 (6)	N1D—N2D—C8D	121.8 (6)
N3B—N2B—C8B	126.7 (6)	N3D—N2D—C8D	128.3 (6)
N4B—N3B—N2B	110.0 (6)	N4D—N3D—N2D	110.1 (6)
N4B—N3B—C14B	123.8 (6)	N4D—N3D—C14D	123.3 (7)
N2B—N3B—C14B	126.2 (7)	N2D—N3D—C14D	126.5 (7)
N3B—N4B—C7B	103.7 (6)	N3D—N4D—C7D	103.3 (6)
C6B—C1B—C2B	119.2 (8)	C6D—C1D—C2D	119.8 (8)
C6B—C1B—H1BA	120.4	C6D—C1D—H1DA	120.1
C2B—C1B—H1BA	120.4	C2D—C1D—H1DA	120.1
C3B—C2B—C1B	121.0 (8)	C3D—C2D—C1D	119.7 (8)
C3B—C2B—H2BA	119.5	C3D—C2D—H2DA	120.1
C1B—C2B—H2BA	119.5	C1D—C2D—H2DA	120.1
C2B—C3B—C4B	119.9 (8)	C2D—C3D—C4D	121.4 (8)
C2B—C3B—H3BA	120.1	C2D—C3D—H3DA	119.3
C4B—C3B—H3BA	120.1	C4D—C3D—H3DA	119.3
C5B—C4B—C3B	119.5 (8)	C3D—C4D—C5D	119.0 (9)
C5B—C4B—H4BA	120.3	C3D—C4D—H4DA	120.5
C3B—C4B—H4BA	120.3	C5D—C4D—H4DA	120.5
C4B—C5B—C6B	121.1 (8)	C4D—C5D—C6D	119.7 (8)
C4B—C5B—H5BA	119.5	C4D—C5D—H5DA	120.1
C6B—C5B—H5BA	119.5	C6D—C5D—H5DA	120.1
C5B—C6B—C1B	119.3 (8)	C1D—C6D—C5D	120.3 (7)
C5B—C6B—C7B	119.5 (7)	C1D—C6D—C7D	119.7 (8)
C1B—C6B—C7B	121.2 (7)	C5D—C6D—C7D	120.0 (7)
N4B—C7B—N1B	112.6 (7)	N1D—C7D—N4D	112.4 (7)
N4B—C7B—C6B	125.0 (7)	N1D—C7D—C6D	124.2 (7)
N1B—C7B—C6B	122.3 (7)	N4D—C7D—C6D	123.4 (7)
C9B—C8B—C13B	123.6 (8)	C9D—C8D—C13D	123.8 (8)
C9B—C8B—N2B	120.0 (7)	C9D—C8D—N2D	118.9 (8)
C13B—C8B—N2B	116.2 (7)	C13D—C8D—N2D	117.3 (7)
C10B—C9B—C8B	117.5 (8)	C8D—C9D—C10D	117.1 (8)
C10B—C9B—H9BA	121.2	C8D—C9D—H9DA	121.5
C8B—C9B—H9BA	121.2	C10D—C9D—H9DA	121.5
C9B—C10B—C11B	120.5 (8)	C11D—C10D—C9D	121.5 (9)
C9B—C10B—H10B	119.8	C11D—C10D—H10D	119.3
C11B—C10B—H10B	119.8	C9D—C10D—H10D	119.3
C12B—C11B—C10B	121.1 (8)	C10D—C11D—C12D	120.4 (9)
C12B—C11B—H11B	119.4	C10D—C11D—H11D	119.8
C10B—C11B—H11B	119.4	C12D—C11D—H11D	119.8
C11B—C12B—C13B	120.3 (8)	C13D—C12D—C11D	120.1 (9)
C11B—C12B—H12B	119.9	C13D—C12D—H12D	119.9
C13B—C12B—H12B	119.9	C11D—C12D—H12D	119.9
C8B—C13B—C12B	117.0 (8)	C12D—C13D—C8D	117.2 (8)
C8B—C13B—H13B	121.5	C12D—C13D—H13D	121.4
C12B—C13B—H13B	121.5	C8D—C13D—H13D	121.4
C19B—C14B—C15B	123.7 (8)	C19D—C14D—C15D	123.9 (7)
C19B—C14B—N3B	118.5 (7)	C19D—C14D—N3D	119.5 (7)
C15B—C14B—N3B	117.8 (7)	C15D—C14D—N3D	116.6 (7)
C16B—C15B—C14B	117.4 (8)	C14D—C15D—C16D	116.9 (8)
C16B—C15B—H15B	121.3	C14D—C15D—H15D	121.6
C14B—C15B—H15B	121.3	C16D—C15D—H15D	121.6
C15B—C16B—C17B	120.7 (8)	C17D—C16D—C15D	121.6 (8)
C15B—C16B—H16B	119.7	C17D—C16D—H16D	119.2
C17B—C16B—H16B	119.7	C15D—C16D—H16D	119.2
C18B—C17B—C16B	120.3 (8)	C16D—C17D—C18D	119.1 (8)
C18B—C17B—H17B	119.8	C16D—C17D—H17D	120.4
C16B—C17B—H17B	119.8	C18D—C17D—H17D	120.4
C17B—C18B—C19B	120.1 (8)	C19D—C18D—C17D	121.3 (8)
C17B—C18B—H18B	120.0	C19D—C18D—H18D	119.3
C19B—C18B—H18B	120.0	C17D—C18D—H18D	119.3
C14B—C19B—C18B	117.8 (7)	C14D—C19D—C18D	117.2 (8)
C14B—C19B—H19B	121.1	C14D—C19D—H19D	121.4
C18B—C19B—H19B	121.1	C18D—C19D—H19D	121.4
			
C7A—N1A—N2A—N3A	−1.8 (8)	C7C—N1C—N2C—N3C	1.6 (8)
C7A—N1A—N2A—C8A	176.8 (7)	C7C—N1C—N2C—C8C	−174.8 (7)
N1A—N2A—N3A—N4A	1.3 (8)	N1C—N2C—N3C—N4C	−1.1 (8)
C8A—N2A—N3A—N4A	−177.3 (7)	C8C—N2C—N3C—N4C	175.0 (7)
N1A—N2A—N3A—C14A	177.8 (7)	N1C—N2C—N3C—C14C	−179.1 (7)
C8A—N2A—N3A—C14A	−0.7 (12)	C8C—N2C—N3C—C14C	−3.0 (12)
N2A—N3A—N4A—C7A	−0.1 (8)	N2C—N3C—N4C—C7C	0.1 (8)
C14A—N3A—N4A—C7A	−176.7 (7)	C14C—N3C—N4C—C7C	178.1 (7)
C6A—C1A—C2A—C3A	−0.9 (12)	C6C—C1C—C2C—C3C	−0.5 (13)
C1A—C2A—C3A—C4A	1.3 (13)	C1C—C2C—C3C—C4C	1.1 (13)
C2A—C3A—C4A—C5A	−0.7 (12)	C2C—C3C—C4C—C5C	−0.8 (13)
C3A—C4A—C5A—C6A	−0.4 (11)	C3C—C4C—C5C—C6C	0.1 (13)
C4A—C5A—C6A—C1A	0.8 (11)	C4C—C5C—C6C—C1C	0.4 (12)
C4A—C5A—C6A—C7A	−174.5 (7)	C4C—C5C—C6C—C7C	−179.8 (7)
C2A—C1A—C6A—C5A	−0.2 (12)	C2C—C1C—C6C—C5C	−0.2 (12)
C2A—C1A—C6A—C7A	175.2 (7)	C2C—C1C—C6C—C7C	−179.9 (8)
N3A—N4A—C7A—N1A	−1.1 (8)	N3C—N4C—C7C—N1C	0.9 (8)
N3A—N4A—C7A—C6A	178.1 (7)	N3C—N4C—C7C—C6C	−178.3 (7)
N2A—N1A—C7A—N4A	1.8 (9)	N2C—N1C—C7C—N4C	−1.5 (8)
N2A—N1A—C7A—C6A	−177.4 (7)	N2C—N1C—C7C—C6C	177.7 (7)
C5A—C6A—C7A—N4A	9.7 (12)	C5C—C6C—C7C—N4C	10.8 (12)
C1A—C6A—C7A—N4A	−165.7 (7)	C1C—C6C—C7C—N4C	−169.4 (7)
C5A—C6A—C7A—N1A	−171.3 (7)	C5C—C6C—C7C—N1C	−168.3 (7)
C1A—C6A—C7A—N1A	13.3 (12)	C1C—C6C—C7C—N1C	11.5 (11)
N1A—N2A—C8A—C13A	65.3 (10)	N1C—N2C—C8C—C13C	−49.9 (10)
N3A—N2A—C8A—C13A	−116.3 (9)	N3C—N2C—C8C—C13C	134.4 (8)
N1A—N2A—C8A—C9A	−110.5 (9)	N1C—N2C—C8C—C9C	123.0 (8)
N3A—N2A—C8A—C9A	67.9 (11)	N3C—N2C—C8C—C9C	−52.6 (11)
C13A—C8A—C9A—C10A	1.8 (14)	C13C—C8C—C9C—C10C	−2.3 (12)
N2A—C8A—C9A—C10A	177.4 (8)	N2C—C8C—C9C—C10C	−174.8 (7)
C8A—C9A—C10A—C11A	−1.2 (15)	C8C—C9C—C10C—C11C	2.6 (13)
C9A—C10A—C11A—C12A	0.5 (15)	C9C—C10C—C11C—C12C	−2.3 (14)
C10A—C11A—C12A—C13A	−0.3 (15)	C10C—C11C—C12C—C13C	1.4 (14)
C9A—C8A—C13A—C12A	−1.7 (13)	C9C—C8C—C13C—C12C	1.5 (12)
N2A—C8A—C13A—C12A	−177.3 (8)	N2C—C8C—C13C—C12C	174.0 (7)
C11A—C12A—C13A—C8A	0.9 (14)	C11C—C12C—C13C—C8C	−1.0 (13)
N4A—N3A—C14A—C19A	−133.8 (8)	N4C—N3C—C14C—C19C	133.0 (8)
N2A—N3A—C14A—C19A	50.1 (11)	N2C—N3C—C14C—C19C	−49.3 (11)
N4A—N3A—C14A—C15A	46.7 (10)	N4C—N3C—C14C—C15C	−45.9 (11)
N2A—N3A—C14A—C15A	−129.5 (8)	N2C—N3C—C14C—C15C	131.8 (8)
C19A—C14A—C15A—C16A	−0.7 (13)	C19C—C14C—C15C—C16C	4.9 (14)
N3A—C14A—C15A—C16A	178.9 (8)	N3C—C14C—C15C—C16C	−176.3 (8)
C14A—C15A—C16A—C17A	−1.1 (13)	C14C—C15C—C16C—C17C	−1.9 (15)
C15A—C16A—C17A—C18A	2.2 (14)	C15C—C16C—C17C—C18C	−0.2 (15)
C16A—C17A—C18A—C19A	−1.4 (13)	C16C—C17C—C18C—C19C	−0.4 (13)
C15A—C14A—C19A—C18A	1.4 (12)	C15C—C14C—C19C—C18C	−5.5 (13)
N3A—C14A—C19A—C18A	−178.2 (7)	N3C—C14C—C19C—C18C	175.7 (7)
C17A—C18A—C19A—C14A	−0.4 (12)	C17C—C18C—C19C—C14C	3.1 (12)
C7B—N1B—N2B—N3B	0.8 (8)	C7D—N1D—N2D—N3D	1.1 (8)
C7B—N1B—N2B—C8B	178.6 (7)	C7D—N1D—N2D—C8D	−173.9 (7)
N1B—N2B—N3B—N4B	0.3 (8)	N1D—N2D—N3D—N4D	−0.9 (8)
C8B—N2B—N3B—N4B	−177.4 (7)	C8D—N2D—N3D—N4D	173.7 (7)
N1B—N2B—N3B—C14B	−178.9 (7)	N1D—N2D—N3D—C14D	−177.3 (7)
C8B—N2B—N3B—C14B	3.5 (12)	C8D—N2D—N3D—C14D	−2.6 (12)
N2B—N3B—N4B—C7B	−1.3 (8)	N2D—N3D—N4D—C7D	0.3 (8)
C14B—N3B—N4B—C7B	177.9 (7)	C14D—N3D—N4D—C7D	176.8 (7)
C6B—C1B—C2B—C3B	−1.6 (14)	C6D—C1D—C2D—C3D	0.3 (12)
C1B—C2B—C3B—C4B	1.6 (14)	C1D—C2D—C3D—C4D	−1.6 (13)
C2B—C3B—C4B—C5B	−1.8 (13)	C2D—C3D—C4D—C5D	0.6 (12)
C3B—C4B—C5B—C6B	2.0 (13)	C3D—C4D—C5D—C6D	1.6 (12)
C4B—C5B—C6B—C1B	−2.0 (12)	C2D—C1D—C6D—C5D	1.8 (12)
C4B—C5B—C6B—C7B	176.7 (8)	C2D—C1D—C6D—C7D	−175.5 (7)
C2B—C1B—C6B—C5B	1.8 (12)	C4D—C5D—C6D—C1D	−2.8 (12)
C2B—C1B—C6B—C7B	−177.0 (8)	C4D—C5D—C6D—C7D	174.6 (7)
N3B—N4B—C7B—N1B	1.8 (9)	N2D—N1D—C7D—N4D	−1.0 (9)
N3B—N4B—C7B—C6B	179.4 (7)	N2D—N1D—C7D—C6D	177.6 (7)
N2B—N1B—C7B—N4B	−1.7 (8)	N3D—N4D—C7D—N1D	0.4 (8)
N2B—N1B—C7B—C6B	−179.3 (7)	N3D—N4D—C7D—C6D	−178.2 (7)
C5B—C6B—C7B—N4B	22.8 (12)	C1D—C6D—C7D—N1D	−2.3 (12)
C1B—C6B—C7B—N4B	−158.5 (8)	C5D—C6D—C7D—N1D	−179.7 (7)
C5B—C6B—C7B—N1B	−159.9 (7)	C1D—C6D—C7D—N4D	176.1 (7)
C1B—C6B—C7B—N1B	18.9 (11)	C5D—C6D—C7D—N4D	−1.3 (12)
N1B—N2B—C8B—C9B	−125.6 (8)	N1D—N2D—C8D—C9D	90.2 (9)
N3B—N2B—C8B—C9B	51.8 (11)	N3D—N2D—C8D—C9D	−83.9 (11)
N1B—N2B—C8B—C13B	50.8 (10)	N1D—N2D—C8D—C13D	−86.3 (10)
N3B—N2B—C8B—C13B	−131.8 (8)	N3D—N2D—C8D—C13D	99.6 (9)
C13B—C8B—C9B—C10B	−1.0 (12)	C13D—C8D—C9D—C10D	1.2 (13)
N2B—C8B—C9B—C10B	175.1 (7)	N2D—C8D—C9D—C10D	−175.1 (8)
C8B—C9B—C10B—C11B	0.1 (13)	C8D—C9D—C10D—C11D	0.9 (14)
C9B—C10B—C11B—C12B	0.2 (14)	C9D—C10D—C11D—C12D	−2.0 (14)
C10B—C11B—C12B—C13B	0.3 (14)	C10D—C11D—C12D—C13D	1.0 (13)
C9B—C8B—C13B—C12B	1.5 (12)	C11D—C12D—C13D—C8D	1.0 (13)
N2B—C8B—C13B—C12B	−174.8 (7)	C9D—C8D—C13D—C12D	−2.1 (13)
C11B—C12B—C13B—C8B	−1.1 (12)	N2D—C8D—C13D—C12D	174.2 (7)
N4B—N3B—C14B—C19B	−116.9 (9)	N4D—N3D—C14D—C19D	137.6 (8)
N2B—N3B—C14B—C19B	62.2 (11)	N2D—N3D—C14D—C19D	−46.4 (11)
N4B—N3B—C14B—C15B	61.9 (11)	N4D—N3D—C14D—C15D	−41.5 (11)
N2B—N3B—C14B—C15B	−119.0 (9)	N2D—N3D—C14D—C15D	134.4 (8)
C19B—C14B—C15B—C16B	−2.7 (15)	C19D—C14D—C15D—C16D	0.0 (13)
N3B—C14B—C15B—C16B	178.6 (9)	N3D—C14D—C15D—C16D	179.1 (8)
C14B—C15B—C16B—C17B	0.2 (16)	C14D—C15D—C16D—C17D	1.0 (14)
C15B—C16B—C17B—C18B	1.8 (16)	C15D—C16D—C17D—C18D	−1.1 (14)
C16B—C17B—C18B—C19B	−1.5 (14)	C16D—C17D—C18D—C19D	0.2 (13)
C15B—C14B—C19B—C18B	3.1 (14)	C15D—C14D—C19D—C18D	−0.8 (13)
N3B—C14B—C19B—C18B	−178.3 (7)	N3D—C14D—C19D—C18D	−179.9 (7)
C17B—C18B—C19B—C14B	−0.9 (13)	C17D—C18D—C19D—C14D	0.7 (13)

### Hydrogen-bond geometry (Å, º)

*Cg*1, *Cg*2, *Cg*3 and *Cg*4 are the centroids of the C1*C*–C6*C*, C8*C*–C13*C*, C1*A*–C6*A* and C1*D*–C6*D* rings, respectively.

**Table:**

*D*—H···*A*	*D*—H	H···*A*	*D*···*A*	*D*—H···*A*
C19*B*—H19*B*···I3	0.95	3.03	3.844 (8)	145
C3*A*—H3*AA*···*Cg*1^i^	0.95	2.87	3.636 (8)	138
C5*A*—H5*AA*···*Cg*2	0.95	2.89	3.547 (9)	127
C16*A*—H16*A*···*Cg*3^ii^	0.95	2.85	3.657 (9)	144
C16*D*—H16*D*···*Cg*4^iii^	0.95	2.96	3.769 (10)	144

Symmetry codes: (i) −*x*+2, −*y*+1, −*z*; (ii) *x*−1, *y*, *z*; (iii) *x*+1, *y*, *z*.
